# Toxicity Investigations of (R)-3-Hydroxybutyrate Glycerides In Vitro and in Male and Female Rats

**DOI:** 10.3390/nu14204426

**Published:** 2022-10-21

**Authors:** Laurie C. Dolan, Alice Raphael Karikachery, Velaphi C. Thipe, Benjamin G. Arceneaux, Kavita K. Katti, Kattesh V. Katti, Alton M. Chesne

**Affiliations:** 1 GRAS Associates, LLC., 11810 Grand Park Avenue, Suite 500, North Bethesda, MD 20852, USA; 2 Department of Radiology, Institute of Green Nanotechnology, University of Missouri, Columbia, MO 65212, USA; 3 Tecton Group, LLC., 370 River Rd., Alexandria, LA 71302, USA

**Keywords:** (R)-3-Hydroxybutyrate glycerides, ketone ester, safety, subchronic, rats, genetic toxicity, beta-hydroxybutyrate, metabolic energy

## Abstract

TCN006, a formulation of (R)-3-Hydroxybutyrate glycerides, is a promising ingredient for enhancing ketone intake of humans. Ketones have been shown to have beneficial effects on human health. To be used by humans, TCN006 must be determined safe in appropriately designed safety studies. The results of a bacterial reverse mutation assay, an in vitro mammalian micronucleus study, and 14-and 90-day repeat dose toxicity studies in rats are reported herein. In the 14- and 90-day studies, male and female Wistar rats had free access to drinking water containing 0, 75,000, 125,000 or 200,000 ppm TCN006 for 92 and 93 days, respectively. TCN006 tested negative for genotoxicity and the no observed adverse effect level (NOAEL) for toxicity in the 14- and 90-day studies was 200,000 ppm, the highest dose administered. In the longer term study, the mean overall daily intake of TCN006 in the 200,000 ppm groups was 14,027.9 mg/kg bw/day for males and 20,507.0 mg/kg bw/day for females. At this concentration, palatability of water was likely affected, which led to a decrease in water consumption in both males and females compared to respective controls. This had no effect on the health of the animals. Although the rats were administered very high levels of (R)-3-Hydroxybutyrate glycerides, there were no signs of ketoacidosis.

## 1. Introduction

Low carbohydrate, ketogenic foods are gaining considerable prominence, as the global demand to mitigate severe adversities of obesity and associated diseases has become a focal point in human health care [1,2,3,4,5,6,7,8,9,10]. Ketogenic foods are specific foods that support an individual in maintaining a state of nutritional ketosis. These generally contain very low carbohydrate, modest protein, and high fat. These foods help to support a ketogenic diet which facilitates the mobilization of endogenous ketones from lipid stores. This combination of foods is expected to induce systemic nutritional ketosis—a process where ketone bodies are produced in vivo serving as alternate energy sources for neurons and various cellular pathways [11,12,13,14,15]. While a plethora of ketone-rich dietary supplements, derivatives of *β*-hydroxybutyrate (BHB), or precursors of BHB (such as medium chain triglycerides, or MCT), are available through various outlets, there is burgeoning interest toward the development of ketone body products (exogenous ketones). Exogenous ketones typically elevate serum and systemic ketone levels [16,17]. There is extensive ongoing research in developing analogs and derivatives of BHB to increase ketone intake of humans. It is important to recognize that BHB is a naturally available ketone that is produced in the human body. Ketones serve as an alternative source of energy to cells, heart, and brain—as a metabolic means to counter situations of carbohydrate deprivation and provide metabolic flexibility [18,19,20,21,22]. Compelling clinical evidence suggests that the monomeric BHB is directly associated with amplification of pleiotropic effects with measurable benefits on the human body for improving metabolic and physical health [16,23,24,25,26].

The mechanism through which ketones act on our body involves production of acetone, acetoacetate, and BHB through downstream metabolic transformation of fatty acids. The high water-solubility of ketone molecules, due to ketogenesis, allows effective transport without the aid of lipoproteins [17,27,28,29,30]. Acetoacetate and BHB are used by the body for energy—BHB is converted to acetoacetate, and acetoacetate is converted back to acetyl-CoA, which enters the citric acid cycle, and subsequently generates ATP. In healthy humans, the body is continually making a small number of ketones to be used by the body for energy; however, this process is upregulated when carbohydrate stores are depleted [31].

Various analogs of ketones continue to present unprecedented opportunities for maintenance of good health [17,25,27,28,29,30,32,33,34,35,36,37,38,39,40,41,42]. Optimum levels of ketones in the body have beneficial effects on brain function [25,36,42]. Increases in plasma ketone levels correlate with brain ketone metabolism and provide a source of energy supply to the brain, helping to mitigate energy crises associated with neurodegenerative diseases which are characterized by a decline in the brain’s glucose metabolism [25,34,36,42]. Systemic presence of ketone molecules is known to promote resistance to oxidative and inflammatory stress and control transcription of genes associated with aging [32,33,34,35,36,38,39,41,42]. Although the effects of a ketogenic diet on exercise performance are controversial, administration of (R)-3-hydroxybutyl (R)-3-hydroxybutyrate ketone ester to trained cyclists has been shown to increase performance [43,44].

As part of ongoing investigations toward developing innovative and safe proprietary approaches for enhancing ketone intake of humans, we have undertaken a detailed toxicological evaluation of TCN006, a proprietary formulation of (R)-3-Hydroxybutyrate glycerides.

## 2. Materials and Methods

### 2.1. Test Substance

The test material used in all studies was TCN006, composed of 86.5% (R)-3-Hydroxybutyrate glycerides, 9.9% glycerol, and 3% (R)-3-hydroxybutyrate. The composition of the test material was confirmed by gas chromatography (GC) and high-performance liquid chromatography (HPLC). Batch 20201202 was used for the genetic toxicity studies and the 14-day range finding study and 20210702 for the 90-day study. 

### 2.2. Bacterial Reverse Mutation Test (Ames Test)

The bacterial reverse mutation test was conducted at Product Safety Labs, Dayton, NJ, USA in accordance with Good Laboratory Practice Regulations and OECD Guideline 471: Bacterial Mutation Test (2020)*. Salmonella (S.) typhimurium* strains TA98, TA100, TA1535, TA1537, and *Escherichia* *(E.) coli* strain WP2 uvrA were used. The main test used the plate incorporation method, both in the presence and absence of metabolic activation, using chemically induced rat liver S9 mix. The S9 mix was sourced from male Sprague Dawley (SD) rats induced with phenobarbital and 5,6-benzoflavone. All bacterial strains and S9 mix were purchased from Molecular Toxicology, Inc., Boone, NC, USA. The test material, TCN006, was prepared in sterile water and sterile water was used as vehicle control. The positive control substances were sodium azide, 9-aminoacridine hydrochloride monohydrate, 2-nitrofluorene, 4-nitroquinolone N-oxide, 2-aminoanthracene, and benzo[a]pyrene. All positive control substances were sourced from Sigma-Aldrich and stored at room temperature, except for 4-nitroquinolone N-oxide, which was stored at 0–20 °C. All positive controls were prepared in dimethyl sulfoxide (DMSO).

In the initial test following the pate incorporation method, the following materials were mixed and poured over a minimal agar plate: 100 µL of prepared test substance, negative (vehicle) control, or positive control, 500 µL of S9 mix or substitution buffer, 100 µL of bacterial suspension (*S. typhimurium* or *E. coli*), and 2000 µL of overlay agar maintained at 45 °C. Concentrations of TN006 were 1.58, 5.0, 15.8, 50, 158, 500, 1580, and 5000 µg/plate. The positive controls in the absence of S9 mix were sodium azide (5 µg/mL) for TA100 and TA1535, 9-aminoacridine hydrochloride monohydrate (500 µg/mL) for TA1537, 2-nitrofluorene (10 µg/mL) for TA98, and 4-nitroquinolone N-oxide (5 µg/mL) for WP2 uvrA. In the presence of S9, the positive controls were 2-aminoanthracene (20 µg/mL) for TA1535 and TA1537, 2-aminoanthracene (100 µg/mL) for WP2 uvrA, and benzo[a]pyrene (50 µg/mL) for TA98 and TA100. Each plate was prepared in triplicate and once the agar gelled, plates were inverted and incubated at 37 °C for an average of about 65 h.

The confirmatory test used the preincubation modification to the plate incorporation test. Test or control substances, bacterial suspension, and S9 or substitution buffers were incubated under agitation for 30 min at 37 °C prior to mixing with overlay agar and proceeding with the remaining protocol described above. All the test materials and concentrations were the same as in the initial tests.

Following incubation, colonies were counted manually, and/or with a plate counter (Colony Plate Reader: Model Colony-Doc-It). Results were considered positive if; (1) the test item (TCN006) showed a substantial increase in colony counts, outside the laboratory historical control range, and (2) the increase is dose related and/or reproducible.

### 2.3. In Vitro Micronucleus Assay

The in vitro micronucleus study was conducted at Integrated Laboratory Systems, LLC (ILS), Morrisville, NC, USA in accordance with Good Laboratory Practice Regulations and OECD Guideline 487: In Vitro Mammalian Cell Micronucleus Test (OECD, 2016), with the exception that analyses of positive control formulations were not performed. The human TK6 lymphoblastoid (TK6) cells used in this assay were sourced from American Type Culture Collection. TK6 cells were cultured in Rosewell Park Memorial Institute (RPMI) 1640 medium, with 10% heat-inactivated horse serum, 1% Pluronic F-68, 0.5% sodium pyruvate, penicillin at 20 units/mL, and streptomycin at 20 µg/mL. Cells were cultured at 37 °C with 6% CO_2_ in the air. Formulations were prepared such that dosing volume does not exceed 10% of the final culture volume. Cells were exposed to test or control material both with and without the addition of S9 activation system. The S9 system was sourced from livers of male SD rats induced by phenobarbital and 5,6-benzoflavone (Molecular Toxicology, Inc., Boone, NC, USA). In treatments including S9, 10% (v/v) liver homogenate with mixed function oxidase was added to cell cultures. The final concentration of liver homogenate in culture medium was 1%.

TK6 cells were exposed to the test article or control substance in 96-well plates in quadruplicate for 4 h with or without S9, or 24 h without S9, at 37 °C and 6% CO_2_ in air. Cells were plated at 2.0 × 10^5^ cells/mL and the final culture volume was 200 µL. The concentrations of TCN006 were 0.313, 0.625, 1.25, 2.5, 5, and 10 mM (corresponding to 55, 111, 223, 445, 890 and 1780 µg/mL). TCN006 was sourced from Baoray Chemical Limited (Hong Kong). Two 2 mL samples of the highest and lowest concentration formulations were analyzed for TCN006 concentration by gas chromatograph. Acceptable results were measured concentrations within ±10% of calculated formulation concentrations and a percent relative standard deviation (%RSD) of ≤10 between the duplicate sample measurements. Results for the formulation used for the 10 mM concentration were within acceptable range for concentration and RSD (5.2% and 5%, respectively) but were slightly lower than the acceptable range for the formulation used for the 0.313 mM concentration (−12.5% and 10%, respectively). This did not affect the validity of the study.

The positive control was cyclophosphamide monohydrate in cells with S9, and vinblastine sulfate in cells without S9. Both positive control substances were sourced from Sigma-Aldrich. Both were dissolved in DMSO, and the final dosing volume did not exceed 1%. The positive controls were used to demonstrate the ability of experimental conditions to detect chromosomal damage. The vehicle control was culture medium containing 10% sterile water (Sigma-Aldrich, St. Louis, MO, USA). The validity of the assay was confirmed by vehicle controls having ≤2% micronucleated cells and positive controls inducing a significant increase in micronucleated cells.

At the end of the culture periods, the cells were analyzed for cytotoxicity and micronucleus induction by flow cytometry. The flow cytometry-based high content cytotoxicity and micronucleus assay was performed using the In Vitro MicroFlow™ kit (Litron Laboratories, Rochester, NY, USA). Sample preparation, staining, and other methods were performed according to manufacturer’s instructions and laboratory standard operating procedures. The data were collected using a validated Becton-Dickinson FACSCantoII™ flow cytometer (BD Biosciences, San Jose, CA, USA). Unless limited by cytotoxicity, 5000 (±500) cells from each sample were analyzed for the frequency of micronuclei.

Micronuclei were identified using a combination of characteristics of size (as measured by light scatter) and fluorescence (based on differential staining steps) that differentiated debris and necrotic and apoptotic cells from “healthy” cells with true micronuclei. Cytotoxicity was measured as relative survival of cells from treated cultures compared to cells from vehicle control cultures using ratios of counted nuclei to counted beads (inert latex microspheres added to each sample). Higher nuclei to bead ratios correspond to increased cell survival. Viable cell counts were also determined from 1 replicate culture at each exposure level on a Tecan Spark multimode plate reader using trypan blue exclusion and the counts were used to calculate relative increase in cell count (RICC) for each test article and positive control exposure. For the 4 h incubations, cytotoxicity as measured by RICC was less than 10% or 20% in the absence or presence of S9, respectively, at all test article concentrations; therefore, all test concentrations were included in the analysis. All concentrations were also included in the analysis of data from the 24 h incubation in the absence of S9, as cytotoxicity under this condition was less than 20%.

### 2.4. 14-Day Range Finding/Palatability Study in Rats

This study was conducted at Product Safety Labs, Dayton NJ, USA in accordance with OECD Guideline 407: Health Effects, Repeated Dose 28-Day Oral Toxicity Study in Rodents (2008) but was 14 days in duration. Product Safety Labs is AAALAC accredited and certified in the appropriate care of all live experimental animals and maintains current staff training ensuring, animals were handled humanely during the experimental phase of this study. In total, 40 IGS Wistar Han rats from Charles River Laboratories were divided into 4 groups (n = 5/sex/group), a vehicle control group and 3 treatment groups. The concentration of TCN006 in the drinking water was 75,000, 125,000 and 200,000 ppm in the treatment groups to target approximate exposures of 8750, 14,583, and 23,333 mg/kg bw/day, based on estimated daily water consumption of 35 g for a 300 g rat. The animals were individually housed in suspended stainless steel cages. Dosing solutions in drinking water were prepared weekly and stored under refrigeration. Results of a preliminary study showed that the test material was stable in drinking water after refrigeration for 7 days followed by ambient temperature storage for 4 days. Drinking water with or without TCN006 was available ad libitum to the rats for 14 days in the treatment and control groups, respectively. Water was refreshed every 4 days and as needed throughout the study to ensure ad libitum access to the test substance at their respective concentrations for a complete 14-day interval. Food was available ad libitum until the night before termination.

Animals were observed at least twice daily for viability and cage-side observations were done daily during the study; all abnormal findings were recorded. On day 0, and weekly thereafter, a detailed observation was conducted. These observations were meant to note any changes in skin, fur, eyes, and mucus membranes, occurrence of secretions and excretions, and autonomic activity. Furthermore, changes in gait, posture, or response to handling, as well as tonic movements, stereotypies, and bizarre behavior were noted. The date and time of all observations was also noted. Animals were weighed on days 0, 3, 7, 10, and 14 to calculate changes in body weight throughout the study. Food consumption was measured in accordance with weight to calculate food efficiency. Water intake was also measured twice per week.

Clinical pathology was performed on all surviving animals, including hematology, clinical chemistry, coagulation, urinalysis, and thyroid hormone analysis. Animals were fasted overnight prior to blood collection from the sublingual vein or abdominal aorta under isoflurane anesthesia. For hematology, about 500 µL of blood was drawn into K_2_ EDTA tubes and stored refrigerated until analysis of the following parameters: Hematocrit, platelet count, reticulocyte count, hemoglobin concentration, red blood cell count, white blood cell count, mean corpuscular hemoglobin, red cell distribution width, differential leukocyte count, and mean corpuscular volume. For coagulation, about 1.8 mL of blood was collected into tubes containing 3.2% sodium citrate and centrifuged to generate plasma. Plasma was stored at −80 °C until analysis of activated partial thromboplastin time and prothrombin time. For clinical chemistry, approximately 1 mL of blood was collected in blank tubes and centrifuged to generate serum, which was stored at −80 °C until analysis. The clinical chemistry parameters were albumin, fasting glucose, aspartate aminotransferase, alkaline phosphatase, globulin, total protein, total bilirubin, inorganic phosphorous, sodium, blood creatinine, high density lipoprotein, low density lipoprotein, sorbitol dehydrogenase, calcium, triglycerides, chloride, potassium, urea nitrogen, total cholesterol, and alanine aminotransferase. For urinalysis, samples were collected at least 15 h prior to blood collection and stored on ice or under refrigeration until analysis of the following parameters: bilirubin, ketone, quality, blood, microscopic urine sediment, specific gravity, color, pH, volume, clarity, total protein, urobilinogen, and glucose.

All animals were euthanized by exsanguination from the abdominal aorta under isoflurane anesthesia. All animals were subjected to full necropsy, and the following organs were weighed: adrenals (combined), kidneys, testes (combined), brain, liver, thymus, epididymis (combined), ovaries with oviducts (combined), uterus, heart, and spleen. Tissues were preserved if indicated by signs of toxicity or target organ involvement.

### 2.5. 90-Day Repeat Dose Study in Rats

This study was conducted at Product Safety Labs, Dayton NJ, USA in accordance with Good Laboratory Practice Regulations and OECD Guideline 408: Health Effects, Repeated Dose 90-Day Oral Toxicity Study in Rodents (2018). As mentioned previously, Product Safety Labs is AAALAC accredited and requires for animals to be handled humanely. This study used 80 IGS Wistar Han rats from Charles River Laboratories and divided them into 4 groups (n = 10/sex/group). The treatment groups received 75,000, 125,000, or 200,000 ppm of TCN006 dissolved in the drinking water. The control group received drinking water with no TCN006. The dose levels in the treatment groups correspond to intakes of 6563, 10,937, and 17,500 mg/kg/day, based on estimated daily water consumption of 35 g for a 400 g rat. The animals were individually housed in suspended stainless steel cages. Animals received ad libitum access to drinking water for the duration of the study and to food up to the night before termination. Males and females received the test substance for 92 and 93 days, respectively. Dosing solutions in drinking water were prepared weekly and stored under refrigeration. Water was refreshed every 4 days and as needed throughout the study to ensure continuous administration of the desired dose for each group. Results of a preliminary study (see Section 2.4) showed that the test material would be stable in the drinking water as administered. Stability and concentration of the test material were confirmed using water samples taken at the beginning, middle, and end of the study.

During the acclimation period, all animals underwent ophthalmologic evaluations including focal illumination, indirect ophthalmoscopy, and slit-lamp microscopy. Mydriatic eyedrops were used and examinations were done under subdued light. All animals were observed at least twice a day, and all observations were recorded. On Day 0, and weekly thereafter, detailed clinical observations were noted, including changes to skin, fur, eyes, and mucus membranes, occurrence of secretions or excretions, and evidence of autonomic activity. Additionally, changes in gait, posture, response to handling, stereotypies, or bizarre behavior were all noted. Following week 11 of the exposure period, all main study animals were subjected to a functional observational battery (FOB). Animals were evaluated for excitability, autonomic function, gait, and sensorimotor coordination. The FOB also evaluated abnormal clinical signs, including convulsions, tremors, unusual behavior, dehydration, and general appearance. Forelimb and hindlimb grip strength and foot splay measurements were also recorded. Motor activity measurements coincided with the FOB and were monitored using an automated Photobeam Activity System (San Diego Instruments, Inc., San Diego, CA, USA) that can handle 20 animals per session. Each animal is evaluated for 1 h over a span of six 10-min phases. Body weights were taken twice during acclimation, on day 0, and weekly afterward in order to calculate changes in body weight. Water consumption was determined twice per week for each animal to confirm consumption of the test material.

Clinical pathology was completed on each surviving animal at the end of the study period (Day 93 for males and Day 94 for females). This included hematology, clinical chemistry, coagulation, urinalysis, and thyroid hormone analysis. All animals were fasted overnight prior to blood draw from the sublingual vein or abdominal aorta under isoflurane anesthesia. All blood draw methods and measured parameters are the same as the 14-day study described above. For thyroid hormone analysis, blood was analyzed for triiodothyronine (T3), thyroxine (T4) and thyroid-stimulating hormone (TSH). All parameters were quantified by ELISA. Vaginal smears were also collected from female to determine stage of estrus at the time of euthanizing.

Animals were euthanized by exsanguination under isoflurane anesthesia. All animals were subjected to gross necropsy, all gross lesions were recorded. The same organs weights were recorded as in the 14-day study described above. The following organs were weighed at least 24 h after preservation in 10% neutral buffered formalin: prostate and seminal vesicles with coagulating gland (combined), thyroid, parathyroid, and pituitary glands. The following organs and tissues were preserved in 10% neutral buffered formalin for possible future histopathological evaluation: accessory genital organs (prostate and seminal vesicles), ileum with Peyer’s patches, rectum, jejunum, salivary glands (sublingual, adrenals kidneys submandibular, and parotid), all gross lesions, larynx, skeletal muscle, aorta, liver, skin, bone (femur), lungs, spinal cord (3 levels: cervical, bone marrow (from femur & mid-thoracic, and lumbar) sternum), lymph node, mandibular lymph node, mesenteric spleen, brain (sections including medulla/pons, cerebellar, and cerebral cortex), mammary gland, sternum, nasal turbinates, stomach, nose, thymus, cecum, ovaries, thyroid, cervix, oviducts, trachea, colon, pancreas (with islets), urinary bladder, duodenum, parathyroid, uterus, esophagus, peripheral nerve (sciatic), vagina, Harderian gland, pharynx, heart, and pituitary gland. The following organs and tissues were preserved in modified David’s fixative and stored in ethanol for possible future histopathological evaluation: eyes, optic nerve, testes, and epididymis. Histological examinations were completed on animals in the control and high dose groups.

### 2.6. Statistical Methods

In the bacterial reverse mutation test, means and standard deviations were calculated for all quantitative data. The data generated in the micronucleus assay was analyzed using the Statistical Analysis System (SAS) version 9.2 (SAS Institute, Cary, NC, USA). Normality of the vehicle control data was determined using the Shapiro–Wilk test and homogeneity was determined using Levene’s test. Normally distributed, homogenous data was analyzed using the one-way analysis of variance (ANOVA). Treatment groups were compared to the appropriate control using Dunnett’s multiple comparison test. Dose-dependent changes were analyzed using a linear regression model. For data that was not normally distributed and homogenous, appropriate non-parametric tests were used. Significance is determined based on *p* < 0.025, and a one-tailed t-test was used to confirm a positive response in cells exposed to a positive control substance (*p* < 0.05). For the 14-day range finding and palatability study, means and standard deviations were calculated for all quantitative data and significance was defined by *p* < 0.05. For all in-life, continuous data, for example body weight or water consumption, two-way ANOVA was used to test the effect of time and treatment. Significant changes observed were further analyzed post hoc by Dunnett’s test of the individual treatment groups and controls. Organ weight data was evaluated for homogeneity and normality. In the case of homogenous and normal data, one-way ANOVA was used. If one-way ANOVA was significant, treatment and control groups were compared using Dunnett’s test. In the case of data that was not homogenous or normal, the Kruskal–Wallis non-parametric analysis of variance was used. If this test was significant, Dunn’s test was used to compare treated groups to controls. The 90-day study used the same statistical methods as the 14-day study. The statistical analysis for the 14- and 90-day studies was designed to determine differences from control rather than differences between treatment groups, in order to determine a no observed adverse effect level (NOAEL). The statistical analysis for body weight, body weight gain, food consumption and food efficiency included analyses for each interval as well as an analysis for all intervals (marginal).

## 3. Results

### 3.1. Mutagenicity

TCN006 did not induce reverse mutation in *S. typhimurium* or *E. coli* in the absence or presence of S9 metabolism, under the reported experimental conditions (Table 1). None of the tested concentrations were toxic or caused precipitation in either experiment. Contamination, which did not obscure the count, was observed in one plate (*E. coli* −S9 at 15.8 µg/plate). Marked increases in revertant numbers were obtained in each experiment following treatment with the positive control items, indicating that the assay systems were functioning correctly. The studies are considered valid because the mean plate counts for untreated control plates fell within the range of historical control data and positive controls induced expected responses.

### 3.2. Micronucleus Study

As shown in Table 2, the average micronucleus frequency in TK6 cells was not statistically different from the concurrent vehicle control at any test concentration, and there was no statistically significant dose response for the 4 h tests in the absence or presence of S9 and the 24 h test in the absence of S9. Micronucleus frequencies at all test article exposure levels were within the laboratory historical vehicle control ranges, and the positive control (vinblastine sulfate) induced a mean percent micronucleus frequency that was statistically increased over the vehicle control under each testing condition. The tests were valid, as the vehicle control cultures for each test condition had average micronucleus cell frequencies of ≤2%, and the positive controls induced a statistically significant increase in micronucleus frequency compared to the vehicle controls in all exposure conditions.

### 3.3. 14-Day Range Finding/Palatability Study in Rats

Exposure to the test substance in drinking water at 75,000, 125,000, and 200,000 ppm for a period of 14-days did not induce any adverse changes in clinical condition, body weight, food or water consumption, hematology, coagulation, clinical chemistry, urinalysis, organ weights or gross pathology of male or female rats. Based on water consumption and body weight, the mean overall (Days 0–14) nominal daily intake of the test substance in rats receiving 75,000, 125,000, and 200,000 ppm of the material in the drinking water was calculated to be 12,639.0, 18,465.7, and 17,681.0 mg/kg bw/day for males and 9678.3, 13,676.0, and 20,016.8 mg/kg bw/day for females, respectively. Based on the results, the same concentrations used for the 14 day study were chosen for the 90-day study.

### 3.4. 90-Day Study

The test substance was determined to be stable under the conditions of storage at PSL over the course of this study. All dose preparations were considered to have met the target concentrations in the dosing mixtures. Analysis indicated that TCN0006, as incorporated in the drinking water, was homogeneously incorporated and stable for up to 10 days. Based on the overall neat test substance stability, homogeneity, and dietary stability in the drinking water, and dose preparation concentration verification results, the animals are considered to have been provided with expected target concentrations of TCN006 (75,000, 125,000 and 200,000 ppm).

**Figure 1:**
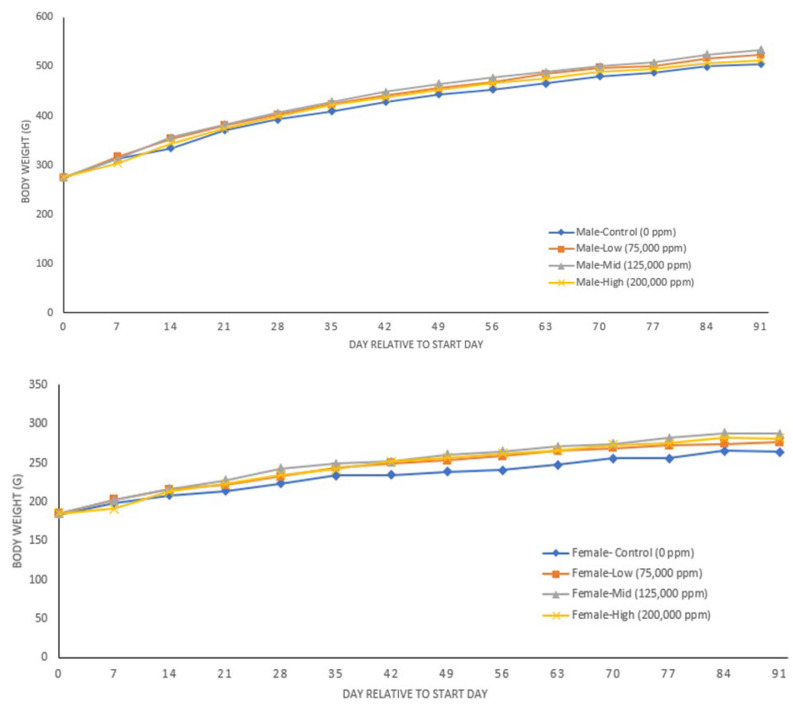
Body weights of male and female rats in the 90-day toxicity study of TCN006.

There were statistically significant decreases in drinking water consumption in all treatment groups when compared to control, particularly in rats receiving 200,000 ppm (Table S1). Mean body weight and daily body weight gain values for all groups of treated male and female rats were comparable to the control group throughout the study (Figure 1 and Table S2). Body weight and water consumption measurements collected throughout the study were used to calculate the mean overall daily intake (Days 0–91) of TCN006. The mean overall daily intake of TCN006 in rats administered 75,000, 125,000, and 200,000 ppm in drinking water was 7042.9, 11,831.1, and 14,027.9 mg/kg bw/day for males and 11,586.4, 14,740.3, and 20,507.0 mg/kg bw/day for females.

Mean daily food consumption values for each group of treated female rats were lower than controls at most time points (*p* < 0.05–0.001); however, there were no significant differences between treated females and controls for food consumption averaged across each time interval (marginal analysis). Conversely, food consumption of treated males was lower than controls only for the marginal analysis. There was no effect of the test substance on feed efficiency of males or females for any interval; however, the marginal analysis showed an increase in food efficiency for each group of treated males and females (Tables S3 and S4).

There were no mortalities throughout the study and there were no test substance-related FOB or motor activity observations. Red staining on the neck or head and ano-genital staining was noted in more treated females than control females; however, incidences were similar among groups and did not have any toxicological correlates. These findings are likely due to the color of the drinking water containing the test substance and feed in concert with stereotypical grooming behavior, and with no other correlated effects, are not considered to be adverse. No other clinical signs were noted. Ophthalmologic examinations of all animals were normal. There were no test substance-related changes in coagulation parameters and the few changes that occurred in hematology (slight decreases in MCHC and increases in RDW of females) were considered unrelated to test substance administration because they occurred sporadically and were of low magnitude. (Table S5). There was no effect of the test material on urinalysis of male or female rats (Table S6).

Study personnel attributed some changes in clinical chemistry to be related to test material administration. Serum concentrations of triglycerides were increased in all groups of treated males and cholesterol and HDL increased in all groups of treated females (Table 3). The changes observed in female cholesterol as well as all increases in male triglycerides levels are within historical control values for this age and strain of rat, and without microscopic correlates, are considered non-adverse. Changes in thyroid hormones were considered unrelated to test substance administration because they occurred sporadically without dose correlation or pathology to the thyroid, parathyroid or pituitary and were of low magnitude. Small increases in serum calcium and protein were noted in some groups of females which were not considered to be test material-related because of minimal magnitude of the change. Some changes occurred in some other clinical chemistry parameters (i.e., decreased ALT in high dose males and low and high dose females, decreased AST in high dose females, decreased ALKP in all groups of treated females, decreased CREA in high dose females) which should not be considered adverse because higher, rather than lower values are indicative of toxicity to the liver, bone or kidneys.

Selected tissues were weighed for organ weight determinations, with little variation of the group means when treated groups were compared to most of the mean organ weights of the same-sex controls, except for kidneys and thyroids in females (Table 4). In high dose females, kidney weights were increased regardless of whether they were reported as absolute values or relative to body or brain weight. In females, thyroid weights of the treated groups were lower than those of controls, regardless of how they were reported. The investigators noted that control females had a mean thyroid weight greater than all male groups, which was unexpected due to lower body weight than the males. This anomaly, plus the larger standard deviation present in the control female thyroid weight data suggested these organ weight data were likely affected by greater variation in trimming of a few glands, which influenced the results. No gross or microscopic observations were found in any organ (including the thyroid and kidney) which were suggestive of any adverse effects of any dose of the test article. All findings observed were incidental and spontaneous changes commonly seen in control rats of this age and strain. There was no effect of the test substance on estrus.

## 4. Discussion

Results of the studies conducted with TCN006 indicate that the substance is not mutagenic or clastogenic in vitro. Based on the results of the 90-day study, the) NOAEL in Wistar rats is 200,000 ppm in drinking water (14,027.9 mg/kg bw/day for males and 20,507.0 mg/kg bw/day for females), the highest concentration administered. At this concentration, palatability of water was likely affected, which led to a decrease in water consumption in both males and females. This had no effect on the health of the animals, as evidenced by normal growth, behavior, hematology, ophthalmology, organ pathology and urinalysis. No clinical signs were noted that were attributed to toxicity. There was no evidence of gastrointestinal distress or signs of or ketoacidosis. Although concentrations of ketones in the urine were highly variable in low- and mid-dose males, a relationship to dose was not apparent. There was a decrease in food consumption at several time points in treated females, a decrease in the marginal mean for food consumption for treated males, and an increase in marginal mean food efficiency in treated males and females; however, there was no effect of the test material on body weight, suggesting that the test material may have been a source of energy for the animals. This finding is consistent with findings of other studies which show that ketones are used as a source of energy when carbohydrate supplies are limited. It is unknown whether the substance affected body composition because this was not measured in the rats.

Clinical chemistry parameters of animals exposed to TCN006 were within or close to normal range; however, there were a few statistically significant changes of note. Increased triglycerides in all groups of treated males (although not statistically significant in mid dose males) and total cholesterol and HDL in all groups of treated females were attributed to test material administration but were not considered adverse. There was no increase in LDL in females; therefore, it is likely that the increase in total cholesterol was due to the increase in HDL, or “good” cholesterol. It is also likely that the increase in triglycerides in treated males is due to metabolism of the glycerol component of the ketone and/or free glycerol in the test material to triglycerides. Glycerol and BHB make up 42.14% and 57.86% of the (R)-3-Hydroxybutyrate glyceride ketone molecule, respectively. Based on the test material containing 86.5% of the ketone, 9.9% glycerol, and 3% β-hydroxybutyrate, male and female rats exposed to 200,000 ppm of the test substance were exposed to approximately 6500 mg/kg bw/day and 9500 mg/kg bw/day glycerol and 7440 mg/kg bw/day and 10,880 mg/kg bw/day BHB, respectively (assuming 100% metabolism of the ketone to glycerol and BHB). TCN006 had no deleterious effects on levels of minerals, glucose or protein in plasma of rats, indicating that at the levels administered, it did not have an adverse effect on the nutritional status of the rats.

As mentioned in CIR (2015), glycerol is rapidly absorbed from the intestine and the stomach, distributed throughout blood and the extracellular space, and excreted renally [45]. The elimination half-life of glycerol is approximately 30–45 min. Most orally administered glycerol is incorporated into body fat or brought into glycolysis or gluconeogenesis pathways (principally in the liver) via glycerokinase-catalyzed phosphorylation and oxidation to dihydroxyacetone phosphate, with ultimate conversion to CO_2_ and water in the former pathway, and glucose and glycogen synthesis in the latter. Glycerol can also combine with free fatty acids to form triglycerides. The results of the current study are consistent with results of studies which show no adverse effects of dietary consumption of up to 20% glycerol (8824 mg/kg bw/day) on the health of rats [46,47]. As demonstrated by the current study, the total amount of glycerol exposure to the rats from the test substance (approximately 6500 mg/kg bw/day and 9500 mg/kg bw/day in males and females) was well tolerated, as evidenced by the fact that only toxically insignificant increases in triglycerides were observed. There also was no evidence of any fatty changes in organ pathology or increases in organ weights that could be attributed to administration of glycerol.

Absorbed BHB is metabolized in the liver to acetoacetate and acetone. Both BHB and acetoacetate are taken up by extrahepatic tissues and are used as energy sources [48]. Acetone produced from BHB may be excreted by the lungs or metabolized to molecules that could be used as energy sources (e.g., pyruvate or acetate). BHB is a metabolic intermediate that constitutes 70% of ketone bodies produced during ketosis [49]. Maximal ketone body production is approximately 185 g/day during times of limited glucose availability such as fasting, indicating that the human body has the capacity to handle large amounts of ketones. The results of the current study are consistent with the results of a 28-day toxicity study in rats which shows safety of D-β-hydroxybutyrate ester diets containing 11.4% D-β-hydroxybutyrate ester (12 and 15 g/kg body weight/day in male and female rats, respectively) [49]. Assuming 100% conversion of D-β-hydroxybutyrate ester to its components BHB and (R)-1,3 butanediol, the Clarke et al. 2012 study [50] supports the safety of 7 g/kg bw/day BHB in rats (BHB accounts for 58.6% of the D-β-hydroxybutyrate ester). Interestingly, in that study, rats in the ketone ester group consumed significantly less feed (similar to what was observed in the current study with (R)-3-Hydroxybutyrate glycerides), but also gained significantly less weight than rats in the control groups (unlike what was observed in the current study). It is likely that in the study with (R)-3-Hydroxybutyrate glycerides, the tendency of the ketone to affect weight gain of rats was mitigated by administration of large amounts of the test material, which provided some caloric value and therefore counteracted the effect of reduced caloric intake from food on body weight.

The results of the studies described in this manuscript show that (R)-3-Hydroxybutyrate glycerides is non-genotoxic as analyzed in a bacterial reverse mutation assay and an in vitro micronucleus study in TK6 cells. They also show that rats tolerate high levels of (R)-3-Hydroxybutyrate glycerides and suggest that the ingredient could be safely consumed by humans at a high level of intake. The NOAEL in a 90-day drinking water study in rats is 200,000 ppm (14,027.9 mg/kg bw/day for males and 20,507.0 mg/kg bw/day for females), the highest concentration administered. Safety factors are applied to NOAELs from studies in rats to extrapolate to safe doses in humans. Typically, a safety factor of 100 is applied to account for extrapolation of data from rats to humans and human variability [51]. However, lower safety factors have been accepted for macroingredients like (R)-3-Hydroxybutyrate glycerides because it is recognized that doses in diets of rats are limited because of the potential to cause nutritional imbalances [52]. Nutritional imbalances were not observed in the current study; however, the doses given to the rats were limited by palatability of the drinking water. For the related substance (R)-3-hydroxybutyl (R)-3-hydroxybutyrate, which was successfully notified as Generally Recognized as Safe (GRAS) to FDA, the safety factor between the NOAEL in rats (12 g/kg bw/day) and the highest estimated intake level in humans (1.2 g/kg bw/day) is 10 [53]. Both glycerol and BHB are well tolerated in humans at higher levels than would be predicted from application of a 100-fold safety factor to their rat NOAELs. The NOAEL for glycerol in a 50-day clinical study in humans of 2200 mg/kg/ bw/day [45] is 4 times lower than the NOAEL in rats mentioned above (8824 mg/kg bw/day). A clinical study showed that BHB is safe in healthy humans at up to 25.50 g/day for 90 days (estimated 364 mg/kg bw/day for a 70 kg individual), which is 5.5 times lower than the rat NOAEL of 2000 mg/kg bw/day [54,55]. The aforementioned clinical study showed a lack of an effect of BHB on markers of nutritional status of participants (i.e., electrolyte and glucose levels in blood, body weight and composition, and bone density), as well as complete blood count, blood pressure, hemoglobin A1c, immune status and psychological well-being.

Based on available information from similar substances, the authors of this manuscript propose that safety factors derived from studies with the similar substances (R)-3-hydroxybutyl (R)-3-hydroxybutyrate, glycerol, and BHB should be taken into consideration when deriving a safe level of intake of (R)-3-Hydroxybutyrate glycerides for humans.

## Figures and Tables

**Table 1:** Reverse mutation assay of TCN006 in *Salmonella typhimurium* and *Escherichia coli*: mean number of revertants/plate.

Concentration (µg/Plate)	TA98		TA100		TA1535		TA1537		WP2uvrA	
	−S9	+S9	−S9	+S9	−S9	+S9	−S9	+S9	−S9	+S9
**Experiment 1**										
0 ^a^	21	29	116	120	11	13	11	15	46	53
1.58	25	29	120	116	14	11	9	15	48	53
5	22	23	122	119	13	12	13	11	53	56
15.8	21	29	116	123	11	13	11	17	43	57
50	22	31	112	111	10	10	16	15	44	56
158	25	29	117	125	11	15	15	10	46	52
500	20	30	110	120	10	12	9	12	47	53
1580	22	29	119	122	12	8	9	16	54	50
5000	24	30	112	109	13	14	13	10	55	50
Positive control	194 ^b^	159 ^c^	519 ^d^	746 ^c^	547 ^d^	492 ^e^	247 ^f^	254 ^e^	612 ^g^	180 ^e^
Historical negative control (−S9, range)	15–35		72–129		6–20		5–17		30–66	
Historical negative control (+S9, range)	18–32		78–123		8–18		8–20		32–68	
**Experiment 2**										
0 ^a^	21	27	119	120	12	12	9	10	46	54
1.58	20	24	109	118	13	12	8	8	50	53
5	18	25	111	117	13	15	8	11	45	59
15.8	21	23	114	120	10	9	9	12	43	62
50	25	25	105	102	10	9	10	11	44	52
158	20	25	111	113	13	11	8	12	42	59
500	21	25	113	107	17	11	10	13	48	50
1580	22	21	102	119	11	11	9	13	49	53
5000	24	21	110	118	11	9	10	10	47	53
Positive control	176 ^b^	168 ^c^	510 ^d^	622 ^c^	521 ^d^	391 ^e^	452 ^f^	248 ^e^	702 ^g^	178 ^e^
Historical negative control (−S9, range)	17–38		80–138		6–22		6–16		26–59	
Historical negative control (+S9, range)	17–38		82–134		6–17		6–14		32–62	

Substance was tested using the standardized plate incorporation assay (Experiment 1) and the pre-incubation method (Experiment 2). Results are means of three replicates per test condition. ^a^ Sterile water vehicle; ^b^ 2-nitrofluorene; ^c^ benzo[a]pyrene; ^d^ sodium azide; ^e^ 2-aminoanthracene; ^f^ 9-aminoacridine; ^g^ 4-nitroquinoline-N-oxide.

**Table 2:** Results of TCN006 in vitro micronucleus study.

Concentration (µg/mL)	% A/N	Nuclei: Beads	% Relative Survival	% MN	*p*-Value	RICC
Four hour incubation without S9						
0 (water)	1.8	9.0	100	0.5	NR	100.0
55	2.2	8.5	94.0	0.5	0.9398	101.7
111	1.6	8.8	97.9	0.6	0.8308	103.9
223	2.1	9.1	100.8	0.4	0.9986	95.8
445	2.0	8.5	94.2	0.7	0.1550	98.1
890	1.6	9.0	99.9	0.6	0.6813	95.5
1780	1.8	8.7	95.9	0.5	0.9270	93.7
				Trend	0.4361	
VIN	15.6	5.1	56.5	14.4 *	<0.0001	98.1
Four hour incubation with S9						
0 (water)	1.7	12.6	100	0.5	NR	100
55	1.3	12.8	101.5	0.4	0.9995	96.7
111	1.4	13.5	107.0	0.5	0.9120	82.2
223	1.4	15.1	120.1	0.4	0.9995	92.9
445	1.3	14.1	112.0	0.6	0.7759	95.1
890	1.1	13.2	105.0	0.6	0.6593	93.9
1780	1.5	15.1	120.2	0.6	0.4419	82.2
				Trend	0.0553	
CP	7.5	8.1	64.2	4.9 *	0.0005	39.7
Twenty four hour incubation without S9						
0 (water)	1.8	11.7	100.0	0.6	NR	100
55	1.6	11.6	98.9	0.5	0.9999	101.3
111	1.8	11.8	101.0	0.4	1.0000	107.8
223	1.6	12.7	108.3	0.4	1.0000	102.5
445	1.5	11.3	96.3	0.4	1.0000	81.9
890	1.5	11.3	96.3	0.6	0.9866	100.1
1780	1.4	13.2	112.4	0.3	1.0000	109.5
				Trend	0.0902	
VIN	11.8	7.2	61.2	12.0 *	<0.0001	59.9

Concentrations reported are nominal and results are presented as means of quadruplicate cultures. %A/N = % apoptotic /necrotic cells; CP = 3.0 µg/mL cyclophosphamide monohydrate MN = micronucleated cells; N = not relevant; RICC = relative increase in cell count; S9 = metabolic activation system; VIN = 5.0 ng/mL or 0.75 ng/mL vinblastine sulfate (short or long term test positive control). * Significant at *p* < 0.05 (Student’s t-test, Statistical Analysis System, version 9.2). Results for test material not significant at any concentration (ANOVA/Dunnett’s for comparison against vehicle control and linear regression modeling for trend test, significance at *p* < 0.025, Statistical Analysis System, version 9.2).

**Table 3:** Clinical chemistry data for the 90-day toxicity study of TCN006 in rats.

Parameter	Control	75,000 ppm	125,000 ppm	200,000 ppm	Historical Control
Males					
AST (U/L)	79.5 ± 22.3	80.5 ± 14.8	77.6 ± 18.9	72.3 ± 10.4	63–175
ALT (U/L)	33.1 ± 7.7	35.1 ± 8.0	33.2 ± 7.7	24.1 ± 3.1 **	19–48
ALKP (U/L)	52.2 ± 10.1	48.0 ± 6.7	49.6 ± 8.9	47.4 ± 10.3	36–141
BILI (mg/dL)	0.068 ± 0.019	0.065 ± 0.012	0.058 ± 0.018	0.053 ± 0.0016	0.04–0.2
BUN (mg/dL)	19.9 ± 3.4	18.4 ± 1.7	18.8 ± 2.9	17.9 ± 3.3	10.7–20.0
CREA (mg/dL)	0.249 ± 0.039	0.224 ± 0.033	0.263 ± 0.050	0.233 ± 0.056	0.3–0.5
CHOL (mg/dL)	70.9 ± 11.3	72.8 ± 15.1	65.1 ± 15.5	64.7 ± 7.5	37–95
LDL (mmol/L)	0.375 ± 0.073	0.342 ± 0.126	0.313 ± 0.120	0.268 ± 0.039	ND
HDL (mmol/L)	1.221 ± 0.258	1.291 ± 0.298	1.145 ± 0.302	1.108 ± 0.146	ND
TRIG (mg/dL)	63.7 ± 18.4	99.8 ± 28.4 **	85.8 ± 25.2	101.2 ± 35.7 **	27–160
SDH (U/L)	15.29 ± 4.31	20.50 ± 8.84	18.70 ± 5.78	14.94 ± 5.19	ND
GLUC (mg/dL)	230.6 ± 48.0	237.3 ± 50.8	259.7 ± 47.7	227.7 ± 49.5	106–184
TP (g/dL)	5.46 ± 0.26	5.56 ± 0.35	5.73 ± 0.29	5.62 ± 0.15	5.6–7.6
ALB (g/dL)	3.90 ± 0.16	3.96 ± 0.23	4.05 ± 0.18	3.94 ± 0.17	3.6–4.7
GLOB (g/dL)	1.56 ± 0.19	1.60 ± 0.23	1.68 ± 0.23	1.68 ± 0.14	1.8–2.5
Ca (mg/dL)	12.02 ± 0.66	11.92 ± 0.72	12.52 ± 0.78	11.78 ± 0.32	9.1–11.9
P (mg/dL)	9.24 ± 0.69	9.27 ± 1.02	10.04 ± 0.80	8.81 ± 0.52	3.64–8.4
Na (mmol/L)	142.2 ± 2.7	141.0 ± 1.9	141.2 ± 2.1	142.0 ± 3.2	137–147
K (mmol/L)	7.16 ± 0.90	7.80 ± 0.97	8.31 ± 1.24 *	6.79 ± 0.82	3.88–6.11
Cl (mmol/L)	103.62 ± 1.70	103.52 ± 2.11	103.20 ± 1.84	103.25 ± 1.59	98–106
TSH (ng/mL)	3.245 ± 0.212	2.827 ± 0.293 *	3.930 ± 0.444 **	3.590 ± 0.595	ND
T3 (ng/mL)	1.365 ± 0.052	1.471 ± 0.097 *	1.684 ± 0.122 ***	1.768 ± 0.085 ***	ND
T4 (ng/mL)	62.818 ± 5.247	57.874 ± 5.593	51.336 ± 4.332 ***	53.748 ± 3.091 ***	ND
Females					
AST (U/L)	108.8 ± 17.0	86.4 ± 26.6 *	88.7 ± 18.3	69.0 ± 11.6 ***	64–222
ALT (U/L)	31.4 ± 8.3	23.5 ± 7.3 *	24.5 ± 4.7	20.1 ± 4.1 ***	14–64
ALKP (U/L)	30.4 ± 10.2	19.6 ± 2.3 **	22.4 ± 6.4 *	18.5 ± 3.9 ***	18–62
BILI (mg/dL)	0.067 ± 0.020	0.070 ± 0.018	0.057 ± 0.016	0.062 ± 0.018	0.07–0.2
BUN (mg/dL)	23.8 ± 3.9	23.0 ± 4.1	22.2 ± 4.7	21.2 ± 4.5	11.7–25.0
CREA (mg/dL)	0.313 ± 0.050	0.289 ± 0.056	0.268 ± 0.046	0.237 ± 0.035 **	0.3–0.6
CHOL (mg/dL)	46.5 ± 12.0	61.7 ± 11.0 *	63.7 ± 12.0 **	68.7 ± 9.9 ***	23–97
LDL (mmol/L)	0.120 ± 0.036	0.144 ± 0.044	0.146 ± 0.040	0.160 ± 0.043	ND
HDL (mmol/L)	1.023 ± 0.266	1.358 ± 0.217 *	1.374 ± 0.255 **	1.477 ± 0.261 ***	ND
TRIG (mg/dL)	40.9 ± 11.9	50.3 ± 9.8	51.2 ± 10.4	55.6 ± 16.5	16–175
SDH (U/L)	13.75 ± 6.31	15.79 ± 7.21	11.36 ± 5.10	11.60 ± 3.76	ND
GLUC (mg/dL)	174.0 ± 50.0	175.5 ± 51.9	190.4 ± 63.8	195.2 ± 28.0	89–163
TP (g/dL)	5.24 ± 0.43	5.78 ± 0.26 *	5.59 ± 0.33	5.73 ± 0.63 *	5.7–8.3
ALB (g/dL)	4.10 ± 0.42	4.57 ± 0.17	4.32 ± 0.34	4.59 ± 0.67	3.7–5.8
GLOB (g/dL)	1.14 ± 0.18	1.21 ± 0.17	1.27 ± 0.09	1.14 ± 0.16	1.6–2.3
Ca (mg/dL)	11.20 ± 0.54	11.98 ± 0.67	12.55 ± 1.25 **	12.65 ± 1.06 **	9.5–12.1
P (mg/dL)	9.12 ± 1.38	9.13 ± 1.00	9.35 ± 1.42	9.23 ± 1.13	4.53–9.51
Na (mmol/L)	138.1 ± 3.7	140.9 ± 1.9	142.0 ± 3.0 *	139.8 ± 2.7	135–146
K (mmol/L)	7.67 ± 1.70	7.33 ± 1.27	6.93 ± 0.91	7.40 ± 1.00	3.37–5.11
Cl (mmol/L)	104.72 ± 2.74	105.67 ± 2.25	107.06 ± 2.58	104.98 ± 1.81	97–106
TSH (ng/mL)	3.316 ± 0.424	2.955 ± 0.191	3.956 ± 0.500 **	3.564 ± 0.463	ND
T3 (ng/mL)	1.579 ± 0.139	1.710 ± 0.299	1.827 ± 0.069 **	1.844 ± 0.177 **	ND
T4 (ng/mL)	59.631 ± 8.321	57.190 ± 9.115	57.918 ± 6.676	53.628 ± 5.312	ND

N = 10/group. Data are presented as mean ± standard deviation (SD). * Significantly different from control, *p* < 0.05; ** Significantly different from control, *p* < 0.01; *** Significantly different from control, *p* < 0.001. ALB = albumin; ALKP = alkaline phosphatase; ALT = alanine aminotransferase; AST = aspartate aminotransferase; BILI = total bilirubin; BUN = urea nitrogen; bw = body weight; Ca = calcium; CHOL = cholesterol; Cl = chloride; CREA = creatinine; dL = deciliter; g = grams; GLOB = globulin; GLUC = glucose; HDL = high density lipoprotein cholesterol; K = potassium; kg = kilogram; L = liter; LDL = low density lipoprotein cholesterol; mg = milligrams; mL = milliliter; mmol = millimoles; Na = sodium; ND = no data; ng = nanograms; P = inorganic phosphorus; ppm = parts per million; SDH = sorbitol dehydrogenase; TP = total protein; TRIG = triglycerides; TSH = thyroid stimulating hormone; thyroxine (T4); tri-iodothyronine (T3); U = units.

**Table 4:** Absolute organ weights (g) and relative organ to body weights (%) for the 90-day toxicity study of TCN006 in rats ^†^.

Parameter	Control	75,000 ppm	125,000 ppm	200,000 ppm
Males				
Terminal body weight (g)	483.6 ± 31.3	500.3 ± 32.3	510.2 ± 35.7	496.3 ± 24.7
Adrenals (g)	0.078 ± 0.011	0.075 ± 0.013	0.075 ± 0.011	0.076 ± 0.018
Adrenals/TBW	0.163 ± 0.027	0.149 ± 0.021	0.146 ± 0.017	0.152 ± 0.032
Brain (g)	2.194 ± 0.073	2.188 ± 0.083	2.159 ± 0.263	2.178 ± 0.115
Brain/TBW	4.551 ± 0.276	4.385 ± 0.259	4.235 ± 0.490	4.397 ± 0.300
Epididymides (g)	1.546 ± 0.109	1.640 ± 0.125	1.666 ± 0.146	1.578 ± 0.198
Epididymides/TBW	3.204 ± 0.258	3.286 ± 0.272	3.283 ± 0.408	3.180 ± 0.366
Heart (g)	1.323 ± 0.099	1.335 ± 0.074	1.398 ± 0.066	1.280 ± 0.076
Heart/TBW	2.736 ± 0.125	2.673 ± 0.140	2.750 ± 0.209	2.581 ± 0.124
Kidneys (g)	3.403 ± 0.304	3.418 ± 0.308	3.560 ± 0.214	3.460 ± 0.229
Kidneys/TBW	7.052 ± 0.659	6.827 ± 0.320	7.005 ± 0.599	6.972 ± 0.315
Liver (g)	12.803 ± 1.164	13.572 ± 1.488	14.248 ± 0.960	13.590 ± 0.673
Liver/TBW	26.441 ± 1.044	27.094 ± 1.962	27.964 ± 1.530	27.415 ± 1.412
Pituitary (g)	0.016 ± 0.006	0.016 ± 0.004	0.017 ± 0.006	0.021 ± 0.004
Pituitary/TBW	0.003 ± 0.001	0.003 ± 0.001	0.003 ± 0.001	0.004 ± 0.001
Prostate + SV + CG (g)	3.580 ± 0.412	3.329 ± 0.665	3.525 ± 0.524	3.242 ± 0.337
Prostate + SV + CG/TBW	0.007 ± 0.001	0.007 ± 0.001	0.007 ± 0.001	0.007 ± 0.001
Spleen (g)	0.897 ± 0.154	0.940 ± 0.142	1.021 ± 0.114	0.937 ± 0.102
Spleen/TBW	1.853 ± 0.275	1.885 ± 0.310	2.003 ± 0.198	1.892 ± 0.227
Testes (g)	3.882 ± 0.192	3.856 ± 0.193	3.905 ± 0.297	3.761 ± 0.168
Testes/TBW	8.044 ± 0.437	7.726 ± 0.467	7.705 ± 0.947	7.586 ± 0.342
Thymus (g)	0.324 ± 0.073	0.333 ± 0.093	0.315 ± 0.060	0.353 ± 0.061
Thymus/TBW	0.667 ± 0.134	0.668 ± 0.188	0.616 ± 0.108	0.709 ± 0.109
Thyroid + PT	0.040 ± 0.010	0.043 ± 0.009	0.043 ± 0.009	0.045 ± 0.013
Thyroid + PT/TBW	0.824 ± 0.236	0.855 ± 0.182	0.843 ± 0.169	0.914 ± 0.268
Thyroid + PT/TBrW	0.0180 ± 0.004	0.0195 ± 0.004	0.020 ± 0.004	0.021 ± 0.006
Females				
Terminal body weight (g)	249.6 ± 20.4	263.7 ± 27.7	270.9 ± 19.2	267.5 ± 20.5
Adrenals (g)	0.100 ± 0.010	0.093 ± 0.008	0.094 ± 0.011	0.101 ± 0.012
Adrenals/TBW	0.401 ± 0.050	0.356 ± 0.042	0.351 ± 0.048	0.381 ± 0.061
Brain (g)	1.996 ± 0.112	1.975 ± 0.065	2.001 ± 0.109	2.032 ± 0.101
Brain/TBW	8.026 ± 0.537	7.571 ± 0.900	7.414 ± 0.589	7.625 ± 0.533
Heart (g)	0.873 ± 0.090	0.875 ± 0.075	0.889 ± 0.059	0.919 ± 0.085
Heart/TBW	3.503 ± 0.286	3.330 ± 0.221	3.285 ± 0.141	3.441 ± 0.252
Kidneys (g)	1.827 ± 0.148	1.925 ± 0.190	2.017 ± 0.177	2.269 ± 0.302 ***
Kidneys/TBW	7.347 ± 0.662	7.334 ± 0.717	7.451 ± 0.486	8.482 ± 0.866 **
Kidneys/TBrW	0.915 ± 0.050	0.975 ± 0.096	1.009 ± 0.084	1.114 ± 0.111 ***
Liver (g)	7.037 ± 0.903	7.519 ± 0.818	7.614 ± 0.610	7.961 ± 0.870
Liver/TBW	28.132 ± 2.089	28.534 ± 1.538	28.153 ± 1.989	29.794 ± 2.744
Ovaries (g)	0.148 ± 0.030	0.137 ± 0.016	0.134 ± 0.020	0.135 ± 0.023
Ovaries/TBW	0.595 ± 0.128	0.521 ± 0.055	0.500 ± 0.093	0.502 ± 0.066
Pituitary (g)	0.020 ± 0.006	0.018 ± 0.003	0.018 ± 0.004	0.020 ± 0.005
Pituitary/TBW	0.008 ± 0.002	0.007 ± 0.001	0.007 ± 0.002	0.007 ± 0.002
Spleen (g)	0.560 ± 0.075	0.574 ± 0.091	0.656 ± 0.118	0.597 ± 0.067
Spleen/TBW	2.245 ± 0.252	2.172 ± 0.198	2.42 ± 0.381	2.231 ± 0.187
Thymus (g)	0.249 ± 0.075	0.218 ± 0.044	0.237 ± 0.071	0.229 ± 0.078
Thymus/TBW	0.987 ± 0.241	0.827 ± 0.136	0.878 ± 0.252	0.853 ± 0.264
Thyroid+ PT	0.048 ± 0.014	0.033 ± 0.008 **	0.036 ± 0.010 *	0.033 ± 0.007 **
Thyroid + PT/TBW	1.946 ± 0.597	1.245 ± 0.260 **	1.349 ± 0.424 **	1.225 ± 0.286 **
Thyroid + PT/TBrW	0.024 ± 0.007	0.017 ± 0.004 **	0.018 ± 0.005 *	0.016 ± 0.003 **
Uterus (g)	0.757 ± 0.363	0.632 ± 0.232	0.648 ± 0.285	0.647 ± 0.254
Uterus/TBW	2.988 ± 1.235	2.408 ± 0.874	2.403 ± 1.066	2.397 ± 0.833

N = 10/group. Data are presented as mean ± standard deviation (SD). Relative organ weights (ratios) presented in the table are times 1000. † Data for organ weight as a % of brain weight is included in the table if absolute organ weight or organ weight as a % of body weight was affected. * Significantly different from control, *p* < 0.05; ** Significantly different from control, *p* < 0.01; *** Significantly different from control, *p* < 0.001. bw = body weight; CG = coagulating gland; g = grams; kg = kilogram; mg = milligrams; PT = parathyroid; ppm = parts per million; SV = seminal vesicles; TBW = terminal body weight; TBrW = terminal brain weight.

## Data Availability

Data supporting the study has been archived at PSL.

## Supplementary Materials

The following supporting information can be downloaded at: https://www.mdpi.com/article/10.3390/nu14204426/s1, Table S1: Water consumption (mL/day) of male and female rats during the 90-day toxicity study of TCN006; Table S2: Mean daily body weight gain rats (g/day) of male and female rats during the 90-day toxicity study of TCN006; Table S3: Mean food consumption (g/day) of male and female rats during the 90-day toxicity study of TCN006 in rats; Table S4: Food Efficiency of male and female rats during the 90-day toxicity study of TCN006 in rats; Table S5: Hematology and coagulation data for the 90-day toxicity study of TCN006 in rats; Table S6: Urinalysis data for the 90-day toxicity study of TCN006 in rats.

## References

[1] Broom G.M., Shaw I.C., Rucklidge J.J. (2019). The ketogenic diet as a potential treatment and prevention strategy for Alzheimer’s disease. Nutrition.

[2] LaFountain R.A., Miller V.J., Barnhart E.C., Hyde P.N., Crabtree C.D., McSwiney F.T., Beeler M.K., Buga A., Sapper T.N., Short J.A. (2019). Extended Ketogenic Diet and Physical Training Intervention in Military Personnel. Mil. Med..

[3] Ma S., Suzuki K. (2019). Keto-Adaptation and Endurance Exercise Capacity, Fatigue Recovery, and Exercise-Induced Muscle and Organ Damage Prevention: A Narrative Review. Sports.

[4] Martin-McGill K.J., Lambert B., Whiteley V.J., Wood S., Neal E.G., Simpson Z.R., Schoeler N.E., on behalf of the Ketogenic Dietitians Research Network (KDRN) (2019). Understanding the core principles of a ‘modified ketogenic diet’: A UK and Ireland perspective. J. Hum. Nutr. Diet..

[5] Mohorko N., Černelič-Bizjak M., Poklar-Vatovec T., Grom G., Kenig S., Petelin A., Jenko-Pražnikar Z. (2018). Weight loss, improved physical performance, cognitive function, eating behavior, and metabolic profile in a 12-week ketogenic diet in obese adults. Nutr. Res..

[6] Muscogiuri G., Barrea L., Laudisio D., Pugliese G., Salzano C., Savastano S., Colao A. (2019). The management of very low-calorie ketogenic diet in obesity outpatient clinic: A practical guide. J. Transl. Med..

[7] Roehl K., Falco-Walter J., Ouyang B., Balabanov A. (2019). Modified ketogenic diets in adults with refractory epilepsy: Efficacious improvements in seizure frequency, seizure severity, and quality of life. Epilepsy Behav..

[8] Stafstrom C.E., Rho J.M. (2012). The Ketogenic Diet as a Treatment Paradigm for Diverse Neurological Disorders. Front. Pharmacol..

[9] Westman E.C., Tondt J., Maguire E., Yancy W.S. (2018). Implementing a low-carbohydrate, ketogenic diet to manage type 2 diabetes mellitus. Expert Rev. Endocrinol. Metab..

[10] Włodarek D. (2019). Role of Ketogenic Diets in Neurodegenerative Diseases (Alzheimer’s Disease and Parkinson’s Disease). Nutrients.

[11] Kolb H., Kempf K., Röhling M., Martin S. (2020). Insulin: Too much of a good thing is bad. BMC Med..

[12] Kolb H., Stumvoll M., Kramer W., Kempf K., Martin S. (2018). Insulin translates unfavourable lifestyle into obesity. BMC Med..

[13] Laffel L. (1999). Ketone bodies: A review of physiology, pathophysiology and application of monitoring to diabetes. Diabetes/Metab. Res. Rev..

[14] Silva B., Mantha O.L., Schor J., Pascual A., Plaçais P.-Y., Pavlowsky A., Preat T. (2022). Glia fuel neurons with locally synthesized ketone bodies to sustain memory under starvation. Nat. Metab..

[15] Weis E., Puchalska P., Nelson A.B., Taylor J., Moll I., Hasan S.S., Dewenter M., Hagenmüller M., Fleming T., Poschet G. (2022). Ketone body oxidation increases cardiac endothelial cell proliferation. EMBO Mol. Med..

[16] Cameron D., Soto-Mota A., Willis D.R., Ellis J., Procter N.E.K., Greenwood R., Saunders N., Schulte R.F., Vassiliou V.S., Tyler D.J. (2022). Evaluation of Acute Supplementation With the Ketone Ester (R)-3-Hydroxybutyl-(R)-3-Hydroxybutyrate (deltaG) in Healthy Volunteers by Cardiac and Skeletal Muscle 31P Magnetic Resonance Spectroscopy. Front. Physiol..

[17] Cuenoud B., Hartweg M., Godin J.-P., Croteau E., Maltais M., Castellano C.-A., Carpentier A.C., Cunnane S.C. (2020). Metabolism of Exogenous D-Beta-Hydroxybutyrate, an Energy Substrate Avidly Consumed by the Heart and Kidney. Front. Nutr..

[18] Bak L.K., Walls A.B., Schousboe A., Waagepetersen H.S. (2018). Astrocytic glycogen metabolism in the healthy and diseased brain. J. Biol. Chem..

[19] Bordone M., Salman M.M., Titus H.E., Amini E., Andersen J.V., Chakraborti B., Diuba A.V., Dubouskaya T.G., Ehrke E., De Freitas A.E. (2019). The energetic brain—A review from students to students. J. Neurochem..

[20] Courchesne-Loyer A., Croteau E., Castellano C.-A., St-Pierre V., Hennebelle M., Cunnane S.C. (2016). Inverse relationship between brain glucose and ketone metabolism in adults during short-term moderate dietary ketosis: A dual tracer quantitative positron emission tomography study. J. Cereb. Blood Flow Metab..

[21] Jensen N.J., Wodschow H.Z., Nilsson M., Rungby J. (2020). Effects of Ketone Bodies on Brain Metabolism and Function in Neurodegenerative Diseases. Int. J. Mol. Sci..

[22] Oyarzabal A., Marin-Valencia I. (2019). Synaptic energy metabolism and neuronal excitability, in sickness and health. J. Inherit. Metab. Dis..

[23] Andreux P.A., Van Diemen M.P.J., Heezen M.R., Auwerx J., Rinsch C., Groeneveld G.J., Singh A. (2018). Mitochondrial function is impaired in the skeletal muscle of pre-frail elderly. Sci. Rep..

[24] Bleeker J.C., Visser G., Clarke K., Ferdinandusse S., de Haan F.H., Houtkooper R.H., Ijlst L., Kok I.L., Langeveld M., van der Pol W.L. (2020). Nutritional ketosis improves exercise metabolism in patients with very long-chain acyl-CoA dehydrogenase deficiency. J. Inherit. Metab. Dis..

[25] Evans M., Cogan K.E., Egan B. (2016). Metabolism of ketone bodies during exercise and training: Physiological basis for exogenous supplementation. J. Physiol..

[26] Puchalska P., Crawford P.A. (2021). Metabolic and Signaling Roles of Ketone Bodies in Health and Disease. Annu. Rev. Nutr..

[27] Abdurrachim D., Woo C.C., Teo X.Q., Chan W.X., Radda G.K., Lee P.T.H. (2019). A new hyperpolarized 13C ketone body probe reveals an increase in acetoacetate utilization in the diabetic rat heart. Sci. Rep..

[28] Al-Zaid N.S., Dashti H.M., Mathew T.C., Juggi J.S. (2007). Low carbohydrate ketogenic diet enhances cardiac tolerance to global ischaemia. Acta Cardiol..

[29] Chu Y., Zhang C., Xie M. (2021). Beta-Hydroxybutyrate, Friend or Foe for Stressed Hearts. Front. Aging.

[30] Han Y.-M., Ramprasath T., Zou M.-H. (2020). β-hydroxybutyrate and its metabolic effects on age-associated pathology. Exp. Mol. Med..

[31] Dhillon K.K., Gupta S. (2022). Biochemistry, Ketogenesis.

[32] Brahma M.K., Ha C., Pepin M.E., Mia S., Sun Z., Chatham J.C., Habegger K.M., Abel E.D., Paterson A.J., Young M.E. (2020). Increased Glucose Availability Attenuates Myocardial Ketone Body Utilization. J. Am. Heart Assoc..

[33] Cahill G.F. (2006). Fuel Metabolism in Starvation. Annu. Rev. Nutr..

[34] Jagadish S., Payne E.T., Wong-Kisiel L., Nickels K.C., Eckert S., Wirrell E.C. (2018). The Ketogenic and Modified Atkins Diet Therapy for Children With Refractory Epilepsy of Genetic Etiology. Pediatr. Neurol..

[35] Newman J.C., Verdin E. (2013). Ketone bodies as signaling metabolites. Trends Endocrinol. Metab..

[36] Poff A.M., Moss S., Soliven M., D’Agostino D.P. (2021). Ketone Supplementation: Meeting the Needs of the Brain in an Energy Crisis. Front. Nutr..

[37] Poplawski M.M., Mastaitis J.W., Isoda F., Grosjean F., Zheng F., Mobbs C.V. (2011). Reversal of Diabetic Nephropathy by a Ketogenic Diet. PLoS ONE.

[38] Puchalska P., Crawford P.A. (2017). Multi-dimensional Roles of Ketone Bodies in Fuel Metabolism, Signaling, and Therapeutics. Cell Metab..

[39] Rojas-Morales P., Pedraza-Chaverri J., Tapia E. (2019). Ketone bodies, stress response, and redox homeostasis. Redox Biol..

[40] Rusek M., Pluta R., Ułamek-Kozioł M., Czuczwar S.J. (2019). Ketogenic Diet in Alzheimer’s Disease. Int. J. Mol. Sci..

[41] Waldman H.S., McAllister M.J. (2020). Exogenous Ketones as Therapeutic Signaling Molecules in High-Stress Occupations: Implications for Mitigating Oxidative Stress and Mitochondrial Dysfunction in Future Research. Nutr. Metab. Insights.

[42] Walsh J.J., Caldwell H.G., Neudorf H., Ainslie P.N., Little J.P. (2021). Short-term ketone monoester supplementation improves cerebral blood flow and cognition in obesity: A randomized cross-over trial. J. Physiol..

[43] Cox P.J., Kirk T., Ashmore T., Willerton K., Evans R., Smith A., Murray A.J., Stubbs B., West J., McLure S.W. (2016). Nutritional Ketosis Alters Fuel Preference and Thereby Endurance Performance in Athletes. Cell Metab..

[44] Harvey K.L., Holcomb L.E., Kolwicz S.C. (2019). Ketogenic diets and exercise performance. Nutrients.

[45] CIR Safety Assessment of Glycerin as Used in Cosmetics. https://www.cir-safety.org/sites/default/files/glycer_122014_FR.pdf.

[46] Stoewsand G.S., Dymsza H.A. (1966). Synthetic sources of calories in the diets of rats and dogs. Proc. Seventh Int. Congr. Nutr..

[47] UK SIDS Initial Assessment Report for SIAM 14: Glycerol, Sponsored by the United Kingdom for OECD SIDS Chemical Program. https://hpvchemicals.oecd.org/ui/handler.axd?id=4b0a2d87-3183-40d4-84f5-0e118c647b19.

[48] Shivva V., Cox P.J., Clarke K., Veech R.L., Tucker I.G., Duffull S.B. (2016). The Population Pharmacokinetics of d-β-hydroxybutyrate Following Administration of (R)-3-Hydroxybutyl (R)-3-Hydroxybutyrate. AAPS J..

[49] Dedkova E.N., Blatter L.A. (2014). Role of β-hydroxybutyrate, its polymer poly-β-hydroxybutyrate and inorganic polyphosphate in mammalian health and disease. Front. Physiol..

[50] Clarke K., Tchabanenko K., Pawlosky R., Carter E., Knight N.S., Murray A.J., Cochlin L.E., King M.T., Wong A.W., Roberts A. (2012). Oral 28-day and developmental toxicity studies of (R)-3-hydroxybutyl (R)-3-hydroxybutyrate. Regul. Toxicol. Pharmacol..

[51] WHO Chemical-Specific Adjustment Factors for Interspecies Differences and Human Variability: Guidance Document for Use of Data in Dose/Concentration-Response Assessment. https://apps.who.int/iris/bitstream/handle/10665/43294/9241546786_eng.pdf;sequence=1.

[52] Borzelleca J.F. (1992). Macronutrient substitutes: Safety evaluation. Regul. Toxicol. Pharmacol..

[53] FDA GRAS Notice (GRN) 515, D-Beta-Hydroxybutyrate Ester. https://www.cfsanappsexternal.fda.gov/scripts/fdcc/index.cfm?set=GRASNotices&id=515&sort=GRN_No&order=DESC&startrow=1&type=basic&search=515.

[54] FDA Gras Notice (GRN) No. 1032, D-β-Hydroxybutyrate. https://www.cfsanappsexternal.fda.gov/scripts/fdcc/?set=GRASNotices&id=1032&sort=GRN_No&order=DESC&startrow=1&type=basic&search=butyrate.

[55] Stefan M., Sharp M., Gheith R., Lowery R., Wilson J. (2020). The Effects of Exogenous Beta-Hydroxybutyrate Supplementation on Metrics of Safety and Health. Int. J. Nutr. Food Sci..

