# Supplementary material for: Toxicity Investigations of (R)-3-Hydroxybutyrate Glycerides In Vitro and in Male and Female Rats
Source: Nutrients. 2022 Oct 21;14(20):4426. doi: 10.3390/nu14204426 (PMC9610502; doi:10.3390/nu14204426)
Supplement: Supplementary file 1 [file nutrients-14-04426-s001.zip › nutrients-1948075-supplementary.pdf]

Table S1. Water consumption (ml/day) of male and female rats during the 90-day toxicity study of TCN006

Day of Study	Control	75,000 ppm	125,000 ppm	200,000 ppm
		**Males**		
0 → 3	39.00 ± 5.69	39.47 ± 9.81	30.27 ± 9.39*	13.47 ± 11.82***
3→ 7	60.05 ± 47.02	37.13 ± 4.27*	33.25 ± 10.75**	33.30 ± 5.27***
7 → 10	43.53 ± 12.27	39.90 ± 5.74	42.90 ± 5.88	32.00 ± 4.86**
10 → 14	61.90 ± 40.19	41.23 ± 5.93	40.65 ± 5.49	37.05 ± 21.23*
14 → 17	46.30 ± 9.81	39.60 ± 5.14	38.97 ± 5.39	28.47 ± 3.87***
17 → 21	52.85 ± 25.80	40.40 ± 13.60	40.78 ± 11.66	28.98 ± 4.18***
21 → 24	49.53 ± 14.10	44.03 ± 7.82	43.93 ± 4.52	30.80 ± 3.95***
24 → 28	47.75 ± 11.82	45.18 ± 16.96	46.23 ± 5.90	32.20 ± 4.54***
28 → 31	47.93 ± 14.06	39.37 ± 7.07	42.87 ± 8.83	29.97 ± 6.15***
31 → 35	56.83 ± 19.57	43.28 ± 7.37	45.08 ± 5.60	32.88 ± 3.94***
35 → 38	51.30 ± 14.89	39.33 ± 8.71	40.97 ± 8.09	30.00 ± 3.46***
38 → 42	50.50 ± 20.25	42.10 ± 7.76	50.80 ± 20.86	30.88 ± 3.54***
42 → 45	51.50 ± 15.99	41.67 ± 5.92	44.23 ± 5.59	29.37 ± 7.33***
45→ 49	53.63 ± 18.14	44.25 ± 6.05	45.28 ± 8.62	31.88 ± 8.56***
49 → 52	48.97 ± 16.07	42.23 ± 8.48	45.70 ± 11.09	27.10 ± 3.67***
52→ 56	48.53 ± 11.84	41.13 ± 7.24	42.00 ± 7.13	32.35 ± 11.63***
56 → 59	56.73 ± 20.96	40.00 ± 5.56	41.83 ± 7.74	29.97 ± 3.88***
59 → 63	49.35 ± 12.16	42.43 ± 13.32	37.63 ± 7.43	30.38 ± 10.68***
63 → 66	52.17 ± 18.35	39.23 ± 6.73	41.60 ± 12.60	27.57 ± 3.55***
66 → 70	51.03 ± 16.09	40.80 ± 6.15	41.80 ± 5.64	28.50 ± 3.32***
70 → 73	52.00 ± 16.55	40.13 ± 9.05	38.73 ± 9.42	29.73 ± 10.29***
73 → 77	53.80 ± 18.63	39.25 ± 6.91	40.83 ± 6.52	28.93 ± 3.10***
77 → 80	50.03 ± 15.49	38.80 ± 7.98	39.73 ± 6.45	26.83 ± 2.53***
80 → 84	56.28 ± 27.28	40.53 ± 7.89	39.25 ± 8.50	28.98 ± 2.91***
84 → 87	52.77 ± 22.57	34.27 ± 7.63*	36.67 ± 8.99	25.83 ± 3.48***
87 → 91	53.50 ± 29.73	35.88 ± 7.11	36.65 ± 8.02	30.40 ± 10.39**
		**Females**		
0 → 3	31.27 ± 7.10	32.23 ± 7.94	23.40 ± 2.65**	16.07 ± 5.80***
3→ 7	60.58 ± 58.32	30.40 ± 9.41*	21.58 ± 4.78***	20.20 ± 6.56***
7 → 10	36.83 ± 8.87	32.10 ± 9.43	26.00 ± 6.36**	23.83 ± 3.79**
10 → 14	47.53 ± 16.19	34.18 ± 12.69*	26.03 ± 3.70***	23.00 ± 3.55***
14 → 17	36.10 ± 7.92	31.63 ± 9.10	27.23 ± 9.91*	22.40 ± 4.01***
17 → 21	54.95 ± 23.95	35.15 ± 10.01*	27.78 ± 6.66***	22.70 ± 3.59***
21 → 24	41.07 ± 10.56	35.50 ± 8.28	31.23 ± 4.90*	25.27 ± 4.67***
24 → 28	52.40 ± 29.21	37.05 ± 7.95	28.40 ± 5.99***	26.85 ± 3.39***
28 → 31	39.80 ± 11.07	36.80 ± 7.67	27.90 ± 5.22**	26.33 ± 3.62***
31 → 35	53.20 ± 12.43	39.55 ± 6.84	33.65 ± 9.24***	27.20 ± 5.12***
35 → 38	38.20 ± 10.08	33.60 ± 8.76	30.97 ± 6.12	24.37 ± 4.04***

38 → 42	71.15 ± 29.77	43.43 ± 17.30	31.60 ± 8.74***	27.15 ± 5.65***
42 → 45	45.80 ± 14.56	37.90 ± 10.69	30.10 ± 10.26**	27.53 ± 4.28**
45→ 49	58.43 ± 23.10	40.63 ± 8.28	32.88 ± 10.14***	27.05 ± 6.06***
49 → 52	49.97 ± 17.17	44.63 ± 14.33	29.07 ± 5.83***	24.67 ± 5.23***
52→ 56	51.30 ± 16.81	42.70 ± 9.66	33.75 ± 12.07**	24.93 ± 5.80***
56 → 59	52.97 ± 21.41	41.73 ± 8.34	32.70 ± 7.35**	25.07 ± 5.76***
59 → 63	58.70 ± 22.20	39.40 ± 7.93*	28.58 ± 5.85***	24.95 ± 5.29***
63 → 66	53.87 ± 18.22	35.67 ± 7.90*	31.03 ± 9.54***	27.27 ± 5.07***
66 → 70	56.53 ± 21.24	40.90 ± 9.46	33.43 ± 8.40**	29.35 ± 6.54***
70 → 73	63.30 ± 33.17	42.10 ± 11.62	30.60 ± 8.56***	25.70 ± 5.13***
73 → 77	61.15 ± 22.52	40.75 ± 9.16	31.23 ± 9.24***	27.35 ± 6.12***
77 → 80	45.13 ± 9.75	39.20 ± 10.70	28.47 ± 5.37***	26.80 ± 5.23***
80 → 84	55.83 ± 20.91	40.18 ± 9.55	31.60 ± 6.82***	26.68 ± 5.16***
84 → 87	48.07 ± 13.97	37.97 ± 9.76	24.20 ± 5.05***	25.93 ± 4.91***
87 → 91	48.48 ± 16.46	36.85 ± 10.88	28.80 ± 8.45**	24.90 ± 4.17***

N = 10/group. Data are presented as mean ± standard deviation (SD). ppm = parts per million
*Significantly different from control, $p<0.05$; **Significantly different from control, $p<0.01$;
***Significantly different from control, $p<0.001$.

Table S2. Mean daily body weight gain (g/day) of male and female rats during the 90-day toxicity study of TCN006

Day of Study	Control	75,000 ppm	125,000 ppm	200,000 ppm
		**Males**		
0 → 7	5.39 ± 1.70	5.99 ± 0.65	5.14 ± 4.12	3.96 ± 1.65
7→ 14	3.04 ± 3.29	5.20 ± 0.65	6.26 ± 2.70	5.69 ± 1.02
14 → 21	5.23 ± 3.42	3.83 ± 0.60	3.79 ± 0.71	4.27 ± 0.58
21 → 28	3.13 ± 0.65	3.27 ± 0.52	3.44 ± 0.79	3.79 ± 0.39
28 → 35	2.44 ± 1.84	2.91 ± 0.88	3.11 ± 1.31	3.07 ± 0.71
35 → 42	2.66 ± 2.47	2.54 ± 0.59	2.91 ± 1.29	2.23 ± 0.45
42 → 49	2.23 ± 1.62	2.20 ± 0.74	2.34 ± 1.16	2.39 ± 0.67
49 → 56	1.41 ± 1.40	1.74 ± 0.80	1.70 ± 1.60	1.76 ± 0.76
56 → 63	1.71 ± 2.33	2.39 ± 0.46	1.73 ± 0.27	1.43 ± 0.79
63 → 70	2.11 ± 1.93	1.71 ± 0.62	1.59 ± 0.76	1.94 ± 0.58
70 → 77	1.14 ± 1.32	0.64 ± 0.53	1.09 ± 1.04	0.93 ± 0.51
77 → 84	1.79 ± 1.18	2.13 ± 0.60	2.33 ± 0.84	1.60 ± 0.68
84→ 91	0.64 ± 0.72	1.01 ± 0.74	1.37 ± 1.10	0.87 ± 0.59
Marginal	2.53 ± 2.36	2.74 ± 1.61	2.83 ± 2.17	2.61 ± 1.59
		**Females**		
0 → 7	2.19 ± 0.82	2.54 ± 0.68	2.49 ± 1.80	0.84 ± 2.68
7→ 14	1.31 ± 1.41	1.84 ± 0.41	1.99 ± 1.27	3.17 ± 1.99
14 → 21	0.81 ± 1.15	0.83 ± 0.62	1.56 ± 1.40	1.34 ± 0.64
21 → 28	1.34 ± 1.48	1.57 ± 0.53	2.24 ± 1.22	1.71 ± 0.72
28 → 35	1.54 ± 1.57	1.63 ± 1.25	0.87 ± 0.82	1.10 ± 0.72
35 → 42	0.06 ± 1.09	0.79 ± 0.72	0.37 ± 1.83	1.30 ± 0.98
42 → 49	0.60 ± 1.03	0.47 ± 0.68	1.31 ± 0.84	0.73 ± 0.56
49 → 56	0.27 ± 2.10	0.84 ± 0.98	0.53 ± 1.18	0.77 ± 0.65
56 → 63	1.00 ± 2.08	0.94 ± 0.91	0.91 ± 1.44	0.59 ± 0.88
63 → 70	1.14 ± 0.89	0.56 ± 0.88	0.51 ± 0.92	1.00 ± 0.64
70 → 77	0.04 ± 1.01	0.41 ± 1.23	1.10 ± 0.80	0.31 ± 0.60
77 → 84	1.41 ± 0.75	0.36 ± 1.62	0.81 ± 0.67	0.94 ± 0.96
84→ 91	-0.20 ± 1.00	0.29 ± 1.76	-0.11 ± 0.96	-0.06 ± 0.97
Marginal	0.89 ± 1.43	1.01 ± 1.18	1.12 ± 1.38	1.06 ± 1.34

N = 10/group, except for marginal, which included N = 130/group. Data are presented as mean ± standard deviation (SD). ppm = parts per million. The marginal value is the mean ± SD for every interval. No significant differences were observed between test groups and the control group ($p > 0.05$).

Table S3. Mean food consumption (g/day) of male and female rats during the 90-day toxicity study of TCN006

Day of Study	Control	75,000 ppm	125,000 ppm	200,000 ppm
		**Males**		
0 → 7	26.47 ± 1.48	23.16 ± 2.05	20.53 ± 3.08	16.24 ± 2.42
7→ 14	26.13 ± 4.08	24.59 ± 1.97	23.17 ± 1.83	20.29 ± 1.70
14 → 21	29.03 ± 2.61	25.34 ± 2.05	23.74 ± 2.35	21.01 ± 1.38
21 → 28	28.80 ± 2.47	25.74 ± 1.86	23.86 ± 1.75	20.91 ± 1.66
28 → 35	28.43 ± 2.51	25.80 ± 2.94	23.71 ± 1.86	20.76 ± 2.99
35 → 42	29.37 ± 2.82	25.07 ± 1.86	23.94 ± 1.76	20.66 ± 1.46
42 → 49	28.97 ± 2.30	25.80 ± 2.07	23.16 ± 2.11	20.94 ± 1.23
49 → 56	29.37 ± 2.79	24.74 ± 1.85	24.09 ± 2.35	21.30 ± 1.29
56 → 63	28.23 ± 4.26	24.93 ± 2.25	22.56 ± 2.01	19.51 ± 1.21
63 → 70	28.66 ± 1.99	24.69 ± 1.96	22.63 ± 2.13	19.79 ± 1.27
70 → 77	28.34 ± 2.09	24.23 ± 2.21	22.90 ± 2.15	20.44 ± 0.93
77 → 84	28.01 ± 2.54	23.96 ± 1.52	22.83 ± 2.30	19.66 ± 0.98
84→ 91	26.64 ± 2.92	23.10 ± 2.59	21.33 ± 1.54	18.31 ± 1.11
Marginal	28.19 ± 2.85	24.70 ± 2.20*	22.96 ± 2.26*	19.99 ± 2.04*
		**Females**		
0 → 7	17.51 ± 1.29	15.47 ± 2.09**	14.23 ± 2.16***	11.46 ± 1.81***
7→ 14	18.40 ± 1.30	15.83 ± 1.70**	16.21 ± 1.00*	14.26 ± 1.30***
14 → 21	17.99 ± 2.05	16.00 ± 2.03	15.90 ± 1.82	14.53 ± 1.40***
21 → 28	18.49 ± 1.40	16.40 ± 2.47*	15.96 ± 3.04**	14.13 ± 1.19***
28 → 35	18.77 ± 1.98	16.01 ± 2.87*	15.41 ± 2.30**	13.76 ± 1.12***
35 → 42	18.34 ± 1.17	16.30 ± 2.16*	14.86 ± 1.56***	14.96 ± 2.10***
42 → 49	19.39 ± 0.89	15.34 ± 1.62***	15.19 ± 1.50***	14.23 ± 0.96***
49 → 56	18.59 ± 2.73	15.94 ± 2.73	15.00 ± 1.54**	14.21 ± 1.21***
56 → 63	18.91 ± 1.35	15.64 ± 1.46***	14.73 ± 1.25***	13.40 ± 0.87***
63 → 70	18.84 ± 1.10	15.20 ± 1.50***	14.63 ± 1.13***	13.54 ± 0.73***
70 → 77	18.91 ± 0.98	15.61 ± 1.26***	15.36 ± 1.74***	13.91 ± 1.15***
77 → 84	19.50 ± 1.51	15.33 ± 1.59***	15.13 ± 1.11***	13.69 ± 1.07***
84→ 91	17.33 ± 1.66	14.33 ± 2.72***	13.83 ± 1.29***	12.61 ± 0.79***
Marginal	18.54 ± 1.62	15.65 ± 2.05	15.11 ± 1.78	13.75 ± 1.48

N = 10/group except for marginal, which included N = 130/group. Data are presented as mean ± standard deviation (SD). The marginal value is the mean ± SD for every interval. ppm = parts per million *Significantly different from control, $p<0.05$; **Significantly different from control, $p<0.01$; ***Significantly different from control, $p<0.001$.

Table S4. Food Efficiency of male and female rats during the 90-day toxicity study of TCN006

Day of Study	Control	75,000 ppm	125,000 ppm	200,000 ppm
		**Males**		
0 → 7	0.204 ± 0.064	0.259 ± 0.027	0.223 ± 0.249	0.237 ± 0.078
7→ 14	0.099 ± 0.140	0.213 ± 0.030	0.273 ± 0.123	0.280 ± 0.039
14 → 21	0.174 ± 0.095	0.151 ± 0.019	0.162 ± 0.037	0.204 ± 0.029
21 → 28	0.108 ± 0.015	0.127 ± 0.019	0.144 ± 0.027	0.181 ± 0.015
28 → 35	0.086 ± 0.066	0.112 ± 0.029	0.131 ± 0.056	0.146 ± 0.023
35 → 42	0.087 ± 0.078	0.102 ± 0.023	0.122 ± 0.055	0.108 ± 0.021
42 → 49	0.078 ± 0.058	0.085 ± 0.029	0.102 ± 0.052	0.114 ± 0.029
49 → 56	0.048 ± 0.047	0.069 ± 0.032	0.070 ± 0.069	0.082 ± 0.033
56 → 63	0.049 ± 0.105	0.096 ± 0.018	0.078 ± 0.016	0.073 ± 0.040
63 → 70	0.074 ± 0.069	0.069 ± 0.024	0.070 ± 0.032	0.097 ± 0.026
70 → 77	0.040 ± 0.046	0.026 ± 0.021	0.047 ± 0.046	0.045 ± 0.025
77 → 84	0.064 ± 0.043	0.090 ± 0.027	0.102 ± 0.034	0.082 ± 0.033
84→ 91	0.026 ± 0.025	0.042 ± 0.029	0.064 ± 0.048	0.047 ± 0.032
Marginal	0.088 ± 0.085	0.111 ± 0.067*	0.122 ± 0.105*	0.130 ± 0.079*
		**Females**		
0 → 7	0.125 ± 0.047	0.163 ± 0.033	0.159 ± 0.134	0.038 ± 0.312
7→ 14	0.068 ± 0.071	0.119 ± 0.035	0.121 ± 0.079	0.223 ± 0.144
14 → 21	0.043 ± 0.064	0.052 ± 0.042	0.096 ± 0.074	0.093 ± 0.047
21 → 28	0.071 ± 0.076	0.096 ± 0.028	0.135 ± 0.047	0.119 ± 0.043
28 → 35	0.076 ± 0.066	0.094 ± 0.059	0.055 ± 0.054	0.078 ± 0.048
35 → 42	0.003 ± 0.059	0.052 ± 0.049	0.019 ± 0.119	0.087 ± 0.070
42 → 49	0.032 ± 0.055	0.030 ± 0.046	0.087 ± 0.054	0.052 ± 0.039
49 → 56	-0.001± 0.132	0.046 ± 0.064	0.034 ± 0.076	0.052 ± 0.042
56 → 63	0.048 ± 0.101	0.060 ± 0.059	0.059 ± 0.090	0.041 ± 0.064
63 → 70	0.060 ± 0.044	0.036 ± 0.057	0.034 ± 0.061	0.073 ± 0.047
70 → 77	0.002 ± 0.056	0.027 ± 0.078	0.073 ± 0.055	0.023 ± 0.044
77 → 84	0.071 ± 0.037	0.020 ± 0.105	0.054 ± 0.046	0.066 ± 0.070
84→ 91	-0.014 ± 0.057	0.003 ± 0.160	-0.010 ± 0.071	-0.006 ± 0.078
Marginal	0.045 ± 0.078	0.061 ± 0.081*	0.071 ± 0.088*	0.072 ± 0.116*

N = 10/group, except for marginal, which included N = 130/group. Data are presented as mean ± standard deviation (SD). The marginal value is the mean ± SD for every interval. ppm = parts per million *Significantly different from control, $p<0.05$.

Table S5. Hematology and coagulation data for the 90-day toxicity study of TCN006 in rats

Parameter	Control	75,000 ppm	125,000 ppm	200,000 ppm
**Males**				
RBC ($10^6/\mu L$)	9.200 ± 0.626	9.194 ± 0.714	9.152 ± 0.474	9.101 ± 0.395
HGB (g/dL)	15.14 ± 0.58	14.85 ± 1.11	14.91 ± 0.90	15.34 ± 0.62
HCT (%)	45.97 ± 1.83	45.86 ± 3.32	45.68 ± 2.92	47.12 ± 1.68
MCV (fL)	50.20 ± 1.94	49.81 ± 1.62	49.92 ± 1.84	51.50 ± 1.34
MCH (pg)	16.53 ± 0.89	16.63 ± 1.16	16.30 ± 0.63	17.15 ± 0.77
MCHC (g/dL)	32.97 ± 0.50	33.02 ± 1.89	32.63 ± 0.56	33.32 ± 1.72
RDW (%)	12.38 ± 0.35	12.64 ± 0.58	12.83 ± 0.41	12.87 ± 0.59
RET ($10^3/\mu L$)	168.22 ± 15.59	174.78 ± 27.27	188.90 ± 28.32	180.61 ± 31.78
PLT ($10^3/\mu L$)	1281.0 ± 221.3	1121.6 ± 265.1	1311.5 ± 254.8	1266.9 ± 293.0
WBC ($10^3/\mu L$)	6.918 ± 1.213	7.461 ± 2.347	8.601 ± 2.536	8.162 ± 2.897
LYM ($10^3/\mu L$)	4.848 ± 1.004	4.895 ± 1.642	6.059 ± 1.999	5.890 ± 2.538
NEU ($10^3/\mu L$)	1.579 ± 0.392	1.954 ± 0.725	1.901 ± 0.491	1.690 ± 0.359
EOS ($10^3/\mu L$)	0.192 ± 0.055	0.218 ± 0.049	0.276 ± 0.194	0.231 ± 0.058
MON ($10^3/\mu L$)	0.238 ± 0.083	0.290 ± 0.137	0.216 ± 0.080	0.263 ± 0.076
BAS ($10^3/\mu L$)	0.026 ± 0.005	0.022 ± 0.019	0.052 ± 0.041	0.033 ± 0.019
LUC ($10^3/\mu L$)	0.032 ± 0.013	0.041 ± 0.022	0.041 ± 0.015	0.038 ± 0.016
APTT (s)	15.06 ± 1.71	15.65 ± 1.27	15.81 ± 1.28	15.81 ± 1.07
PT (s)	9.80 ± 0.18	9.78 ± 0.26	9.87 ± 0.21	9.89 ± 0.10
**Females**				
RBC ($10^6/\mu L$)	8.052 ± 1.480	8.477 ± 0.427	8.261 ± 0.551	8.138 ± 0.536
HGB (g/dL)	14.22 ± 2.63	14.81 ± 0.55	14.63 ± 1.03	14.49 ± 0.80
HCT (%)	42.22 ± 7.94	46.03 ± 1.70	44.33 ± 3.10	44.17 ± 2.14
MCV (fL)	52.40 ± 1.40	53.64 ± 1.85	53.56 ± 1.78	54.30 ± 1.35
MCH (pg)	17.65 ± 0.53	17.34 ± 0.82	17.85 ± 1.04	17.80 ± 0.54
MCHC (g/dL)	33.70 ± 0.34	32.32 ± 0.65***	33.32 ± 1.14*	32.79 ± 0.52***
RDW (%)	11.01 ± 0.26	11.33 ± 0.66	11.45 ± 0.32*	11.44 ± 0.39*
RET ($10^3/\mu L$)	145.65 ± 33.25	151.04 ± 36.02	184.43 ± 39.77	163.39 ± 36.47
PLT ($10^3/\mu L$)	1083.9 ± 501.0	1352.4 ± 285.5	1327.3 ± 241.4	1236.8 ± 310.3
WBC ($10^3/\mu L$)	3.577 ± 1.419	3.833 ± 1.222	4.367 ± 1.757	3.865 ± 1.383
LYM ($10^3/\mu L$)	2.515 ± 1.208	2.738 ± 1.030	3.175 ± 1.564	2.802 ± 1.011
NEU ($10^3/\mu L$)	0.816 ± 0.303	0.815 ± 0.287	0.935 ± 0.270	0.804 ± 0.320
EOS ($10^3/\mu L$)	0.146 ± 0.095	0.146 ± 0.066	0.115 ± 0.073	0.116 ± 0.042
MON ($10^3/\mu L$)	0.072 ± 0.066	0.103 ± 0.052	0.106 ± 0.050	0.114 ± 0.057
BAS ($10^3/\mu L$)	0.013 ± 0.012	0.014 ± 0.012	0.019 ± 0.013	0.011 ± 0.010
LUC ($10^3/\mu L$)	0.015 ± 0.008	0.015 ± 0.008	0.019 ± 0.012	0.018 ± 0.012
APTT (s)	14.34 ± 1.94	15.52 ± 0.80	14.83 ± 1.96	14.74 ± 1.64
PT (s)	9.43 ± 0.41	9.70 ± 0.41	9.85 ± 0.34	9.78 ± 0.40

N = 10/group. Data are presented as mean ± standard deviation (SD). *Significantly different from control, $p<0.05$; ***Significantly different from control, $p<0.001$. APTT = activated partial thromboplastin time; BAS = basophils; bw = body weight; dL = deciliter; EOS = eosinophils; fL = femtoliter; HCT = hematocrit;

HGB = hemoglobin; kg = kilogram; LUC = large unstained cells; LYM = lymphocytes; MCH = mean corpuscular hemoglobin; MCHC = mean corpuscular hemoglobin concentration; MCV = mean corpuscular volume; mg = milligrams; MON = monocytes; NEU = neutrophils; pg = picograms; PLT = platelets; ppm = parts per million; PT = prothrombin time; RBC = erythrocytes; RDW = red blood cell distribution width; RET = reticulocytes; s = seconds; WBC = white blood cells (leukocytes); μL = microliter.

Table S6. Urinalysis data for the 90-day toxicity study of TCN006 in rats

Parameter	Control	75,000 ppm	125,000 ppm	200,000 ppm
**Males**				
Urine volume (mL)	11.10 ± 9.45	13.95 ± 9.58	10.65 ± 8.46	16.15 ± 9.17
pH	7.00 ± 0.71	6.95 ± 0.50	7.30 ± 0.86	7.15 ± 0.41
Glucose (mg/dL)	0.00 ± 0.00	0.00 ± 0.00	0.00 ± 0.00	0.00 ± 0.00
Ketones (mg/dL)	4.0 ± 2.1	7.0 ± 12.5	11.5 ± 11.8	1.5 ± 2.4
Protein (mg/dL)	22.5 ± 10.6	28.0 ± 27.2	43.5 ± 39.4	22.5 ± 7.9
Specific Gravity	1.017 ± 0.008	1.014 ± 0.009	1.016 ± 0.009	1.011 ± 0.003
Urobilinogen (EU/dL)	0.20 ± 0.00	0.20 ± 0.00	0.20 ± 0.00	0.20 ± 0.00
**Females**				
Urine volume (mL)	5.12 ± 8.29	4.62 ± 4.91	2.74 ± 3.05	4.44 ± 4.89
pH	6.60 ± 0.77	6.30 ± 0.26	6.56 ± 0.46	6.44 ± 0.58
Glucose (mg/dL)	0.00 ± 0.00	0.00 ± 0.00	0.00 ± 0.00	0.00 ± 0.00
Ketones (mg/dL)	1.5 ± 2.4	2.0 ± 2.6	2.2 ± 5.1	0.0 ± 0.0
Protein (mg/dL)	52.0 ± 42.3	29.0 ± 39.2	52.8 ± 46.0	24.4 ± 31.2
Specific Gravity	1.023 ± 0.010	1.021 ± 0.010	1.022 ± 0.008	1.021 ± 0.009
Urobilinogen (EU/dL)	0.20 ± 0.00	0.20 ± 0.00	0.20 ± 0.00	0.20 ± 0.00

N = 10/group for all groups except N = 9/group for 125,000 and 250,000 ppm females. Data are presented as mean ± standard deviation (SD). No significant differences were observed between test groups and the control group ($p > 0.05$). dL = deciliter; EU = Ehrlich unit; mg = milligrams; ppm = parts per million.
